# Treatment strategies in Alzheimer’s disease: a review with focus on selenium supplementation

**DOI:** 10.1007/s10534-016-9959-8

**Published:** 2016-08-16

**Authors:** Jan Aaseth, Jan Alexander, Geir Bjørklund, Knut Hestad, Petr Dusek, Per M. Roos, Urban Alehagen

**Affiliations:** 1 Department of Research, Innlandet Hospital Trust, Brumunddal, Norway; 2 Department of Public Health, Hedmark University of Applied Sciences, Elverum, Norway; 3 Norwegian Institute of Public Health, Oslo, Norway; 4 Norwegian University of Life Sciences (NMBU), Ås, Norway; 5 Council for Nutritional and Environmental Medicine, Mo i Rana, Norway; 6 Department of Neurology and Center of Clinical Neuroscience, Charles University in Prague, 1st Faculty of Medicine and General University Hospital in Prague, Prague, Czech Republic; 7 Institute of Environmental Medicine, IMM, Karolinska Institutet, Nobels väg 13, Box 210, 17177 Stockholm, Sweden; 8 Department of Clinical Physiology, St.Goran Hospital, Stockholm, Sweden; 9 Division of Cardiovascular Medicine, Department of Medical and Health Sciences, Linköping University, Linköping, Sweden

**Keywords:** Alzheimer’s disease, Copper, Iron, Neuroinflammation, Transmitters, Selenium

## Abstract

Alzheimer’s disease (AD) is a neurodegenerative disorder presenting one of the biggest healthcare challenges in developed countries. No effective treatment exists. In recent years the main focus of AD research has been on the amyloid hypothesis, which postulates that extracellular precipitates of beta amyloid (A*β*) derived from amyloid precursor protein (APP) are responsible for the cognitive impairment seen in AD. Treatment strategies have been to reduce A*β* production through inhibition of enzymes responsible for its formation, or to promote resolution of existing cerebral A*β* plaques. However, these approaches have failed to demonstrate significant cognitive improvements. Intracellular rather than extracellular events may be fundamental in AD pathogenesis. Selenate is a potent inhibitor of tau hyperphosphorylation, a critical step in the formation of neurofibrillary tangles. Some selenium (Se) compounds e.g. selenoprotein P also appear to protect APP against excessive copper and iron deposition. Selenoproteins show anti-inflammatory properties, and protect microtubules in the neuronal cytoskeleton. Optimal function of these selenoenzymes requires higher Se intake than what is common in Europe and also higher intake than traditionally recommended. Supplementary treatment with N-acetylcysteine increases levels of the antioxidative cofactor glutathione and can mediate adjuvant protection. The present review discusses the role of Se in AD treatment and suggests strategies for AD prevention by optimizing selenium intake, in accordance with the metal dysregulation hypothesis. This includes in particular *secondary* prevention by selenium supplementation to elderly with mild cognitive impairment.

## Introduction

Alzheimer’s disease (AD) is a neurodegenerative disorder prevalent in old age. In developed countries 13 % of people over 65 suffer from AD according to the Alzheimer’s Association. Projected AD prevalence indicate 100 million patients globally by 2050 (Alzheimer’s 2015), leading to considerable economic burden for society and suffering for patients and caregivers. AD is classified as genetic or sporadic. Genetic AD is an early-onset hereditary disease representing 1–2 % of diagnosed cases (Campion et al. 1999). In genetic AD mutated genes coding for amyloid precursor protein (APP; chromosome 21) are found, and presenilin 1 (PS1; chromosome 14) and presenilin 2 (PS2; chromosome 1), promote amyloid beta (A*β*) formation. The vast majority of patients suffer from sporadic AD.

Many sporadic AD patients are carriers of the e4 allele of the ApoE gene (apolipoprotein E; chromosome 19). The mechanism whereby ApoE e4 allele increases AD risk is largely unknown (Hardy and Selkoe 2002). Recent research has unmasked minor mutations which mediate an intermediate AD risk. Most genes associated with AD roughly cluster within three metabolic pathways: lipid metabolism, inflammatory response, and endocytosis (Giri et al. 2016). Aging is considered the principal risk factor for sporadic AD, followed by hypertension, dyslipidemia, metabolic syndrome and diabetes (Drachman 2014).

In the present paper, we first discuss treatment strategies based on traditional hypotheses of AD pathogenesis: (a) the transmitter hypothesis, (b) the metabolic hypothesis, (c) the tau protein hypothesis, and (d) the amyloid cascade hypothesis. Then we address the metal-based hypothesis of neuroinflammation which opens new therapeutic possibilities (Ward et al. 2014). Oxidative stress from copper (Cu) and iron (Fe) toxicity is implicated in the metal hypothesis of AD pathogenesis. In this context we discuss a putative therapeutic or preventive role of selenium (Se) supplementation, evoked by a Swedish study reporting improved vitality and reduced signs of inflammation and oxidative stress after Se yeast and coenzyme Q_10_ intervention in an elderly population (Alehagen et al. 2013).

## Traditional hypotheses

### The transmitter hypothesis

Cholinergic neurons projecting to the hippocampus in the temporal lobe are affected early in AD. Deficient spatial memory in rodents has been mapped to grid cells that collect sensory signals in the entorhinal cortex (Hafting et al. 2005). The language problems and declining verbal recall characterizing AD patients are presumed to arise from dysfunction in hippocampal regions (Lim et al. 2012), and these cells are influenced by cholinergic modulation (Konishi et al. 2015). Loss of cholinergic inputs to the hippocampus is a well characterized abnormality in AD. Decreased acetylcholine release combined with reductions in nicotinic and muscarinic receptors in the cortex and hippocampus of AD brains examined post-mortem has been seen (Tata et al. 2014). Acetylcholinesterase inhibitors used in AD treatment act by increasing acetylcholine bioavailability at synaptic loci. Unfortunately, these enzyme inhibitors are not capable of reversing AD, nor slowing disease progression (Wallace and Bertrand 2013). Memantine acts on the N-methyl-D-aspartate (NMDA) receptors blocking glutamate activity (Parsons et al. 2007). A dysfunction of glutamatergic transmission has been hypothesized to be involved in the neurodegeneration in AD. Memantine appears to improve this dysfunction, and has been associated with a moderate decrease in clinical deterioration, with a small positive effect on cognition (Areosa et al. 2005). However, although transmitter dysfunction is seen in AD, it can be suspected that the initial biochemical lesions involve structural and functional impairment of vital proteins responsible for transmitter transport and neuronal integrity.

### The metabolic hypothesis

Clinical studies suggest that the metabolic syndrome, including hypertension, obesity, and insulin resistance or type 2 diabetes (T2DM), is a significant risk factor for AD development (Kivipelto et al. 2005). Disturbed hippocampal insulin signalling is likely present in AD (Hokama et al. 2014). Increased insulin resistance and oxidative stress with elevated levels of advanced glycation end products (AGE) are proposed mechanisms by which metabolic syndrome may increase the risk of AD (Li et al. 2012). A recent study in mice indicated an association between amount of hypothalamic beta-amyloid fragments, neuroinflammation and peripheral glucose intolerance (Clarke et al. 2015). Reactive oxygen species (ROS) and tumor necrosis factor alpha also contribute to this intriguing syndrome combination (Lourenco et al. 2013). As the molecular mechanisms in AD and in insulin resistance seem related, it is tempting to assume that drugs used for T2DM treatment e.g. the glitazones could be protective also in AD. A phase II trial with rosiglitazone for 6 months reported improvements in memory and attention in patients who did not possess an e4 allele of the ApoE gene (Risner et al. 2006), but a phase III rosiglitazone trial failed (Gold et al. 2010). However, insulin resistance is associated with increased AGE formation, decreased protection against oxygen radicals (Aaseth and Stoa-Birketvedt 2000) and raised levels of methylglyoxal (MGA) (Thornalley et al. 1999). These substances are all neurotoxic and possess high reactivity toward thiol (SH) groups such as the numerous microtubule SH groups in the neuronal cytoskeleton. Increased MGA concentrations in cerebrospinal fluid have been reported in AD (Kuhla et al. 2005), and may contribute to tau disintegration and tangle formation.

### The tau hyperphosphorylation hypothesis

**Fig. 1:**
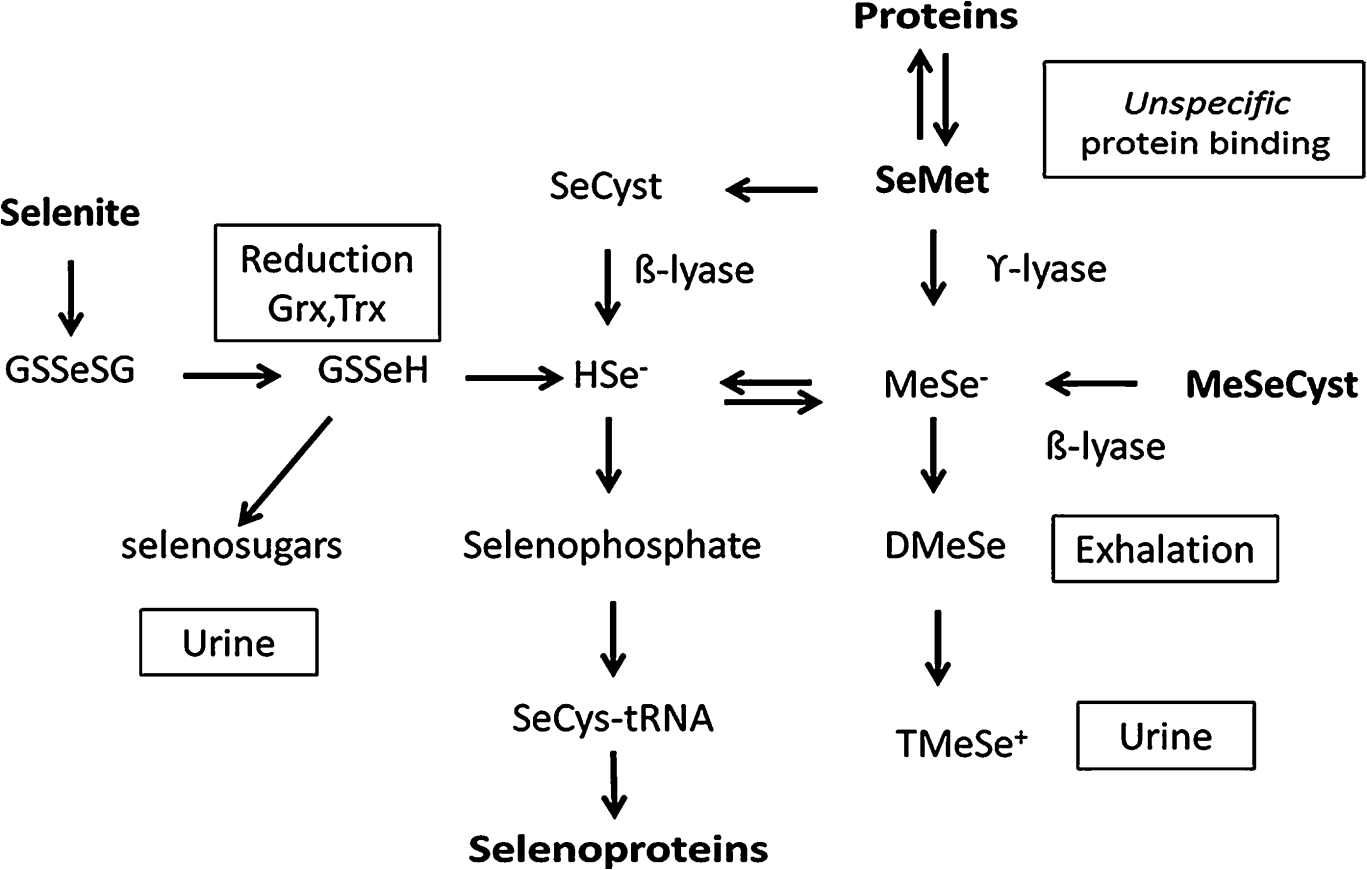
Biotransformation of selenite and seleno-amino acids to selenide, selenoproteins and excretable metabolites. The reduction of selenite is facilitated by GSH, glutaredoxins, glutathione reductase and/or thioredoxin (Trx) and TrxR. It consumes reducing equivalents, NADPH. Selenate that is transported into cells by an anion transport mechanism (Jager et al. 2016), is also reduced to selenide, but the intracellular reduction of selenate to selenite is less efficient and not fully understood. Hydrogen selenide (HSe^−^) and methyl selenide (MeSe^−^) react with oxygen and thiols and complete the redox-cycle. Selenium can be incorporated specifically into selenoproteins via pathways from selenide and the synthesis of selenophosphate. Selenomethioneine can be converted to MeSe^−^ or to selenocysteine via the transsulfuration pathway or unspecifically replace its sulfur analogue in proteins. Among excretory metabolites are selenosugars and at high doses dimethylselenide and trimethyl selenonium ion (TMSe^+^) (Alexander 2014)

Tau is a neuronal, microtubulus-associated protein, which in healthy brains regulates microtubuli dynamics (Yuraszeck et al. 2010). Derangements of microtubuli and of the neuronal cytoskeleton provide clues to the understanding of AD pathogenesis. Intact microtubuli are involved in transport of essential substances from neuronal bodies to synaptic structures. Phosphorylation regulates tau protein binding to microtubuli. Under physiological conditions the tau protein remains soluble, but hyperphosphorylation compromises its normal functions (Mehta et al. 2015), and leads to formation of insoluble neurofibrillary tangles, which are bundles of paired helical protein filaments. Such excessive phosphorylation in AD must result from an imbalance between phosphorylating kinases and de-phosphorylating phosphatases. Increased expression of active kinases adjacent to neurofibrillary tangles has been described in AD (Hochgrafe et al. 2015). One of these kinases and a potential drug target is cyclin-dependent kinase 5 (CDK5). Increased intracellular calcium in AD brains is associated with CDK5 activation (Shukla et al. 2012). CDK5 inhibitors have demonstrated neuroprotective properties in in vitro and in vivo AD models (Zimmer et al. 2012). Sodium selenate also reduces tau phosphorylation, both in cell cultures and in AD mouse models (Corcoran et al. 2010b). Administration of selenate to rodents produces cognitive improvements and reduced neurodegeneration (van Eersel et al. 2010). In these models selenate is presumably converted to specific selenoproteins including glutathione peroxidases (Fig. 1), which may attenuate the intracellular burden of ROS and thereby protect microtubuli in the cytoskeleton.

### The amyloid cascade hypothesis

The amyloid precursor protein (APP) is a glycosylated protein that is uniformly found in cell membranes, most abundant in the brain. A hypothesis claims that the membrane bound protein APP acts as a Cu chaperone, thereby exerting cytoprotective functions (Prohaska and Gybina 2004). Apparently, APP is involved in synaptic repair and in cell signaling (Priller et al. 2006). Substances synthesized in the cell bodies of neurons must be transported outward to the distal synapses. It has been found that APP can mediate interactions that facilitate this transport (Jonsson et al. 2012). A mutation in APP makes the protein more resistant against degradation and protects against cognitive decline (Jonsson 2013). APP is degraded into several peptides by the three intracellular enzymes: α-, β-, and γ-secretases. Soluble cleavage products might also have cytoprotective effects on synaptic structures. Several isoforms of the Aβ-peptide degradation product exist (Mawuenyega et al. 2013). In dominant genetic forms of AD, the disease is thought to be due to over production of Aβ, or an increase in Aβ42 to Aβ40 ratio (Kumar-Singh et al. 2006). The Aβ-peptide with 42 amino acids, Aβ (1–42), usually called Aβ, is an insoluble variant that is prevalent in both sporadic and genetic AD, and constitutes the amyloid core in the precipitated plaques (Gaggelli et al. 2006).

*Active immunization (vaccination)* with either Aβ (1–42) or smaller Aβ fragments has been evaluated in transgenic mouse models of AD. Such vaccination will generally activate the phagocytotic capacity of microglia. The early human tests using a full-length Aβ with an added adjuvant resulted in serious adverse events including aseptic meningoencephalitis (Gilman et al. 2005). Later vaccines were composed from a shorter Aβ fragment in an attempt to avoid side effects. A vaccine denoted CAD106 has reached the clinical phases of development (Wiessner et al. 2011), showing specific antibody response in a majority of treated patients without serious adverse reactions, but without significant therapeutic effect. Other vaccines are in preclinical stages (Panza et al. 2014).

*Passive immunization* involves i.v. administration of antibodies directed specifically against A*β*. Studies in transgenic animals have shown that passive immunization reduces cerebral amyloid load. Bapineuzumab and solanezumab are monoclonal antibodies against A*β* fragments (Tayeb et al. 2013). Both drugs have reached advanced stages of clinical development (Salloway et al. 2014), but without producing significant clinical improvement in humans (Tayeb et al. 2013). So far, immunotherapy has not proven successful in arresting the cognitive decline in AD patients. Since APP and its physiological degradation products exert cytoprotective functions, immunotherapy is not expected to become a treatment of choice.

### The neuroinflammation hypothesis

As discussed above, the insoluble APP derivative A*β* appears to be responsible for plaque formation (Castello and Soriano 2014), and A*β* may induce oxidative stress and microinflammation. An early hypothesis was that suppressing of inflammation could arrest precipitation of A*β* and cognitive decline. This therapeutic exploration began with the observation that several nonsteroidal anti-inflammatory drugs (NSAIDs) decreased A*β* levels in animal models. Ibuprofen, sulindac and flurbiprofen were considered as promising drugs (Du et al. 2014b). The mechanism of action of NSAIDs was ascribed to their inhibition of cyclooxygenases leading to reduced inflammation and thus reduced A*β* precipitation. Yet ibuprofen was ineffective for AD treatment in clinical trials (Pasqualetti et al. 2009), and independent research has failed to show positive results of treatment with NSAIDs in AD. Interestingly however, some NSAIDs possess copper-chelating properties (Puranik et al. 2016) and further research on possible therapeutic effects of selected NSAIDs in relation to the metal hypothesis of neuroinflammation is justified. Also the key role of microglia in neuroinflammatory processes deserves further attention (Xiang et al. 2006). It has been found that microglia surrounds A*β* plaques (ElAli and Rivest 2016). Furthermore, it has been reported that Se abrogates stress-induced microglial cell migration (Dalla Puppa et al. 2007). Further research is necessary to explore if Se attenuates the inflammatory cascade associated with cognitive decline in AD.

## The metal-based hypothesis of neuroinflammation

### Copper and iron dysregulation in AD

It has been reported that Fe and Cu accumulate in AD plaques, and this deposition appears to promote the progression of the Aβ cascade (Altamura and Muckenthaler 2009). Inside of neurons Fe and Cu binding to hyperphosphorylated tau protein precede the formation of intracellular tangles (Barnham and Bush 2014). The presence of free Fe(II) or Cu(I) species will induce deleterious Fenton reactions with ROS generation and microinflammation (Ward et al. 2015).

Experiments indicate that ceruloplasmin (CP), a Cu containing enzyme with ferroxidase activity, protects CNS from Fe(II)-mediated injury (Patel et al. 2002). Torsdottir et al. (2011) found that the CP ferroxidase activity was lower in mild cognitive impairment (MCI) patients than in controls although CP concentrations were similar in both groups (Torsdottir et al. 2011). They explained the discrepancy by a deficient CP Cu incorporation, while the synthesis of apo-CP was unaffected. Brewer et al. (2010) found that CP activity but not amount of CP was lower in AD patients than in controls (Brewer et al. 2010). Here, it is pertinent to recapitulate clinical and neuropathological findings in aceruloplasminemia, a rare hereditary disorder caused by mutation in the CP gene and characterized by absent serum CP activity with Fe deposition in the brain leading to neuropsychiatric symptoms (Kono 2013). Although aceruloplasminemia patients presented five to ten times higher brain tissue Fe concentrations than controls (Morita et al. 1995), no brain amyloid or tau protein precipitation was seen (Gonzalez-Cuyar et al. 2008; Kaneko et al. 2012). Cognitive symptoms in aceruloplasminemia patients include executive dysfunction suggesting fronto-striatal involvement, rather than hippocampal impairment (Kono 2013).

Sparks and Schreurs (2003) reported that minor (0.12 mg/L) Cu excess in drinking water together with cholesterol in the chow for 10 weeks accelerated the formation of amyloid deposits around cerebral vessels and induced learning deficits in a rabbit model (Sparks and Schreurs 2003). Subsequently, Squitti et al. (2011) argued that the level of free serum Cu in AD patients has a predictive value in assessing disease progression (Squitti et al. 2011). When discussing the Cu hypothesis for AD, some characteristics of Wilson disease (WD) may be relevant (Brewer et al. 2010). In WD, Cu levels in the brain are increased by a factor of 5–10 (Horoupian et al. 1988), but the characteristic AD pathology is not present (Meenakshi-Sundaram et al. 2008). Cognitive testing in WD reveals deficit in the executive domain rather than in episodic memory (Iwanski et al. 2015). Together with the findings in aceruloplasminemia, this absence of AD pathology in WD brains suggests that an overall elevation of Cu and Fe concentrations in CNS is not sufficient to initiate Aβ and tau precipitation. Thus, the initiation of Aβ and tau related pathology must be caused by other stimuli, e.g. by long-term exposure to ROS or AGE, although trapping of Cu and Fe may enhance the progression.

Surprisingly, the brains of AD patients are not characterized by increased overall Cu concentrations (Exley et al. 2012; Schrag et al. 2011). However, a hypothesis claims that Cu is removed from various regions of the brain and trapped by Aβ in the pathological plaques. Consistent with this, Maynard et al. (2002) reported that overexpression of the carboxyl-terminal fragment of APP with copper-binding Aβ-fragment in a transgenic mouse model resulted in a redistribution of Cu in the brain and progression of amyloid precipitation (Maynard et al. 2002). Total Cu remains unchanged in serum or cerebrum in AD patients when compared to healthy subjects (Bucossi et al. 2011), but its intracerebral distribution appears to be deranged.

### Metal chelation as a therapeutic strategy

Dyshomeostasis of Cu and Fe in AD brains with accumulation of these metals in plaques and tangles may be accompanied by increased generation of ROS and *progression* of tissue damage. An early study showed that Fe chelation with deferoxamine (125 mg i.m. twice daily/5 days/week for 24 months) resulted in a significant reduction in the rate of decline of daily living skills in 48 AD patients, but not in AD patients receiving placebo (McLachlan et al. 1993). Since then, only few chelating agents have been examined in clinical trials for the treatment of AD, viz. clioquinol (iodochlorhydroxyquin) and PBT2 (5,7-dichloro-2-(dimethylamino)-methyl)-8-hydroxyquinoline). Both agents bind local excesses of Cu and Fe in the brain, thereby presumably retarding the amyloid plaque progression (Bush 2002; Lannfelt et al. 2008; Ritchie et al. 2003). Although none of these studies showed clear clinical effect of chelation therapy in AD, post hoc analyses appeared promising (Barnham and Bush 2014; Faux et al. 2010), and indicated that the hydroxyquinoline derivatives act as chaperone-mimetic agents (Ayton et al. 2015). However, long-term use of hydroxyquinolines may give rise to serious side effects, and the search for less toxic agents is encouraged (Meade 1975).

### Selenium as a protective and chelating agent

Selenium is a trace element crucial to cerebral functions. During Se depletion brain Se is maintained at the expense of other tissues whereas severe Se deficiency causes irreversible brain injury (Burk and Hill 2009). The circulating Se transporter, selenoprotein P (SEPP), appears to have a special role in the delivery of Se to the brain and neurons by entering via the multifunctional apolipoprotein E receptor 2 (ApoER2), a member of the lipoprotein-receptor family that is expressed in neurons in the brain (Burk et al. 2014). In the brain SEPP is primarily provided by synthesis in the astroglial cells. Mice without the machinery for SEPP synthesis under Se deficient conditions develop spasticity, abnormal movements, and seizures (Schweizer et al. 2004). While SEPP is the important extracellular selenoprotein, glutathione peroxidases (GPx1 acting on soluble cytosolic peroxides and GPx4 acting on membrane bound phospholipid peroxides) are important intracellular antioxidants in neurons and glia (Mitozo et al. 2011; Zhang et al. 2010). Also thioredoxin reductases are abundantly expressed in neurons and glia (Godoy et al. 2011). These selenoproteins contain Se in the form of amino acid selenocysteine (SeCys) which differs from cysteine by a single atom (Se vs. S), conferring a lower pKa (5.2 vs. 8.3) and higher reactivity to its functional selenol group. GPx contains only one SeCys residue, whereas SEPP contains 10 SeCys residues conferring a high chelator affinity to Cu(I) (Aaseth et al. 2016). In vitro SEPP has been shown to inhibit Cu induced Aβ aggregation (Du et al. 2014b).

Evidence from human studies suggests a role for Se and selenoproteins in protection against cognitive decline. In the InCHIANTI cohort study of 1012 Italian participants aged 65 years or older, 59 performance-based assessment scores of coordination as well as the MMSE-score were significantly reduced in participants with low plasma Se (<66.7 μg/L) compared to those with higher (>82.3 μg/L) concentrations (Shahar et al. 2010). In the French EVA cohort of 1166 people aged 60–70 years (Berr et al. 2000) a 58 % increased odds ratio of cognitive decline was recorded over four years in participants with a Se concentration in the 1st quartile (<75.8 μg/L) at baseline, as compared to a mean baseline plasma Se level of 86.9 μg/L. Furthermore, cognitive decline was significantly associated with the magnitude of plasma Se decrease over nine years, which attained a decrease of 0.35 μg/L in one of the subgroups (Akbaraly et al. 2007). In a cross-sectional survey of 2000 rural Chinese adults aged 65 years or older, low nail Se concentration was significantly associated with low cognitive scores in four of five tests, with a dose-response effect across Se quintiles (Gao et al. 2007). From Spain Gonzalez-Domınguez et al. (2014) in a cross sectional study found lower Se levels in serum from AD patients (121 μg/L) in comparison to elderly MCI subjects (127 μg/L) (Gonzalez-Dominguez et al. 2014). Also in patients with very mild AD (MMSE score > 20) Se levels in plasma were reported to be lower (82.2 vs. 93.2 μg/L) compared to healthy age-matched elderly subjects in a Dutch cross-sectional study (Olde Rikkert et al. 2014).

**Fig. 2:**
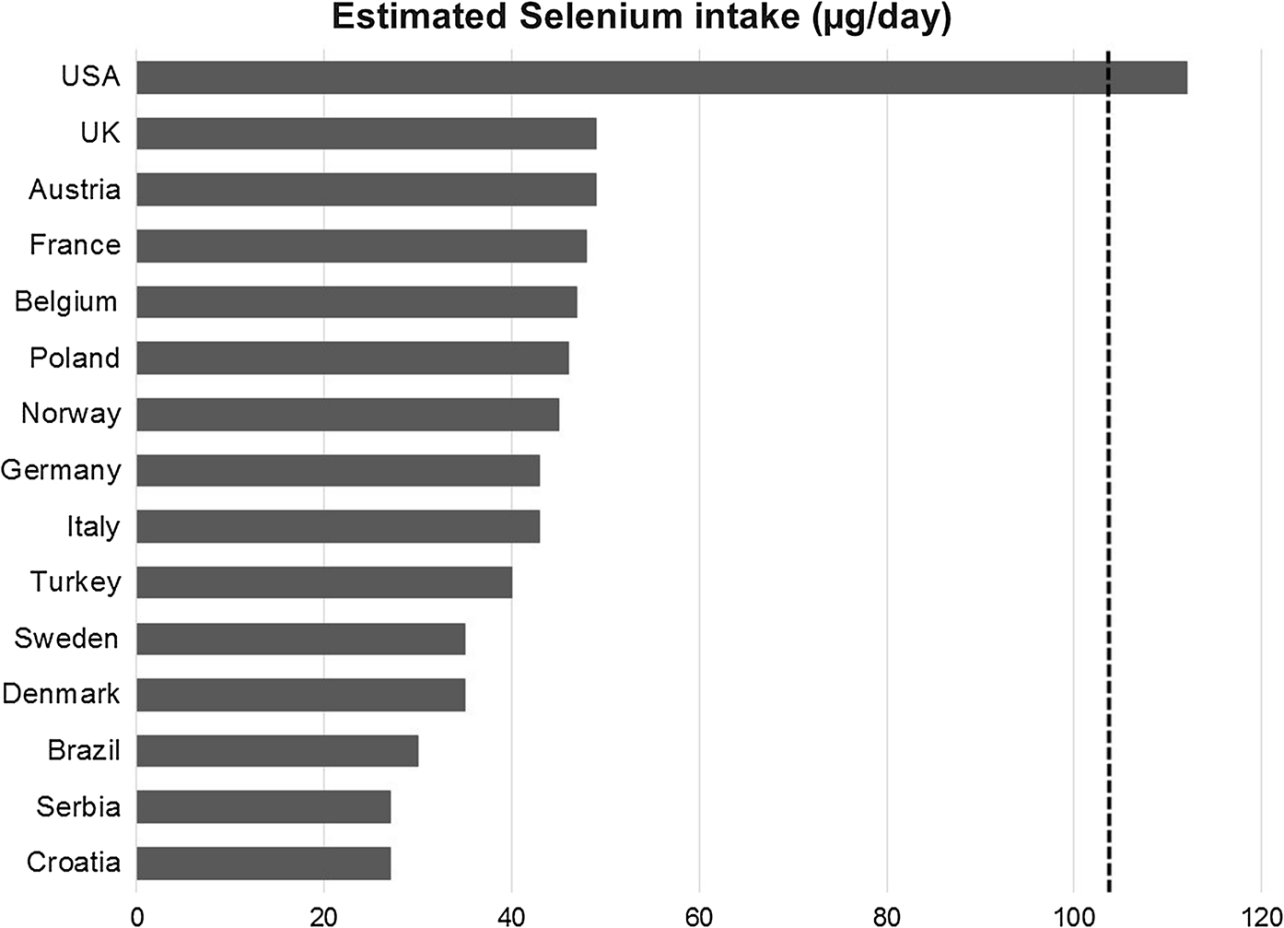
Average daily selenium intake in various countries. Data are from Birgisdottir et al. (2013), Ellingsen et al. (2009), Fairweather-Tait et al. (2011), Vanderlelie and Perkins (2011); Rayman (2005), Maihara et al. (2004), Stoffaneller and Morse (2015). Because of import of wheat, Norway is slightly higher in Se intake than Denmark and Sweden. Optimization of selenoprotein P requires a daily intake of about 105 µg (Hurst et al. 2010), indicated by a dotted line

In several European countries the daily Se intake is too low to obtain optimal function of important selenoenzymes (Fig. 2). The European data on increased cognitive decline at low Se status are in agreement with cross-sectional studies from Brazil and Turkey (Cardoso et al. 2010; Vural et al. 2010). On the other hand a study from India did not find low Se concentrations (174 vs. 188 μg/kg) in patients with AD compared with healthy controls, but their Se levels were higher than the European levels (Krishnan and Rani 2014). Supplementation with compounds containing Se has shown potential for stimulating cognitive improvement (Kesse-Guyot et al. 2011; Scheltens et al. 2010). Cardoso et al. (2015) reported that the daily supplement with one Brazilian nut, corresponding to about 280 µg Se/day, over 6 months was associated with cognitive performance improvement when given to patients with mild cognitive impairment (Cardoso et al. 2015).

Animal models and in vitro Se studies are in accordance with the observations from human surveys. Sodium selenate treatment reduced tau phosphorylation, by activation of protein phosphatase 2A (PP2A), both in cell cultures and tau transgenic animal models (Corcoran et al. 2010b; van Eersel et al. 2010). This treatment prevented and reversed memory and motor deficits; neurofibrillary tangles formation and neurodegeneration in transgenic animals (Ishrat et al. 2009; van Eersel et al. 2010) at a dose of 1 mg/kg bw selenate (van Eersel et al. 2010). Treatment of transgenic AD mice with selenomethionine resulted in reduced total and phosphorylated tau, lower inflammatory biomarkers and improvement in cognition (Song et al. 2014).

### A hypothesis for protection by selenoproteins

High extracellular SEPP levels of have been found in the brain (Bellinger et al. 2008; Steinbrenner and Sies 2013; Takemoto et al. 2010). All regions of mouse brain appear to be dependent on SEPP for maintenance of proper functions (Nakayama et al. 2007). Knock-out of SEPP or ApoER2 in mice resulted in neurological dysfunction, particularly when fed a low Se diet, and it appears that under low Se supply these two proteins are necessary to maintain Se in the brain and prevent neuron degeneration (Burk et al. 2014; Caito et al. 2011; Steinbrenner and Sies 2013; Valentine et al. 2008). Regional progression of neurodegeneration in the brain of the SEPP knock-out mice has been studied in order to map neuronal cell death, and evaluate neuronal structural changes within the hippocampus (Caito et al. 2011). Neurodegeneration was found to be present in all studied brain regions in the knock-out animals fed the Se-deficient diet (Caito et al. 2011). The neurodegeneration was predominantly axonal, however neuronal bodies in the somatosensory cortex and lateral striatum appeared also to be severely deteriorated. Morphological analysis of the hippocampus revealed decreased dendritic length, density and functionally. A defect in the long term potentiation of the hippocampus, essential for memory imprinting, was also noted. These findings are in line with the hypothesis that Se deficiency contributes to functional deficits seen in AD (Caito et al. 2011; Peters et al. 2006).

The expression of SEPP in postmortem tissue from individuals with the hallmark lesions of AD and individuals without these lesions has been examined (Bellinger et al. 2008). SEPP immunoreactivity was co-localized with A*β* plaques and neurofibrillary tangles (Bellinger et al. 2008). These observations suggest some form of interaction between SEPP and A*β*, leading to complex formation. Like SEPP, A*β* is also a strong metal chelator, binding for instance Cu (2016; Ma et al. 2006; Myhre et al. 2013; Syme et al. 2004), and Fe (Myhre et al. 2013). Ternary complexes can be formed between metal cations, A*β* and SEPP and such complexes are presumably less toxic than A*β*-metal complexes alone. Since Cu is one of the metals abundant in A*β* (Myhre et al. 2013) and Cu**(**I**)** binds very strongly to Se atoms, a ternary complex between Cu(I), A*β* and SEPP can explain the co-localization of SEPP with A*β* in AD (Aaseth et al. 2016). SEPP also contains two His-rich regions that contribute to its affinity for Cu and Fe.

**Fig. 3:**
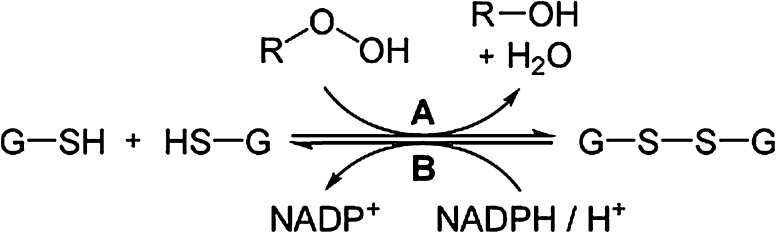
Intracellular detoxification by GPx and GSH. Toxic peroxides (R–OOH) are reduced to non-toxic R–OH by the action of the selenoenzymes glutathione peroxidases (A) in the presence of adequate amounts of the co-factor GSH found intracellularly, in contrast to the negligible GSH-levels found extracellularly. GSH is oxidized to its disulphide GSSG in this reaction. The reduced form GSH is regenerated by intracellular glutathione reductase (B) in the presence of NADPH_2_. This same co-factor, GSH, can also detoxify the compounds glyoxal and methylglyoxal which are neurotoxic byproducts of glucose metabolism, particularly in cases of insulin resistance. The latter reaction requires the presence of the glyoxalase enzyme system (Aaseth et al. 2016)

Thus SEPP chelation blocks metal-mediated Aβ-aggregation and ROS generation [110]. The trapping of SEPP by A*β*-plaques may reduce its availability for the synthesis of intracellular selenoproteins including thioredoxin reductase (TrxR) and GPx. Together with glutathione (GSH) these intra-neuronal selenoenzymes operate as intracellular antioxidants, thereby inhibiting tau aggregation (Du et al. 2014a). Selenium treatment has been reported to reduce tau phosphorylation in transgenic rats (Yim et al. 2009). In healthy brains the microtubule-associated tau protein regulates microtubule dynamics (Yuraszeck et al. 2010). The exact role of GPx and its cofactor GSH for protection of microtubules has yet not been fully elucidated. It should be emphasized here that microtubules are essential parts of the cytoskeleton, thereby maintaining the three-dimensional structure of the neurons. Microtubules play crucial roles in a variety of cellular events, including axonal and dendritic transport and neuronal growth and differentiation (Lasek 1981). Each tubulin monomer has at least 13 free SH groups, and it presumably needs protection against oxidative derangement by the GPx-GSH-system (Fig. 3). The intracellular protector GSH (reduced form) can be optimized by N-acetylcysteine supplementation. A study on AD patients supplemented with N-acetylcysteine over a six month period reported improved performance on memory tests (Adair et al. 2001).

Apparently, optimal function of GPx and SEPP is necessary for protection against the cognitive decline characterizing AD. Optimal function requires higher intakes of Se than officially recommended in Nordic and other European countries. Selenium intake in North-America and some other regions of the world are considerably higher than in Europe (Fig. 2), which might contribute to inconsistencies in the clinical evidence as to the role of Se supplementation (Du et al. 2016; Loef et al. 2011). Results from a Swedish study published in 2015 indicated improved vitality and overall score of health related quality-of-life as a result of intervention with Se and coenzyme Q_10_ to an elderly population with mean baseline plasma Se of 67 μg/L (Johansson et al. 2015). The same intervention study also demonstrated a decreased inflammatory activity as registered by the biomarkers sP-selectin and hs-CRP (Alehagen et al. 2015). The Se supplement dose in this latter study was 200 μg/day, given as selenized yeast. Another intervention that is in progress is the PREADVISE study carried out in the same regions of America as the SELECT study. Thus, the population under investigation has a baseline Se intake that is substantially higher than European intakes (Fig. 3). Participants included in PREADVISE had reported memory complaints at inclusion. The design was double-blind, placebo controlled and randomized, transformed into an observational cohort after discontinuation of supplementation in the SELECT parent trial. PREADVISE participants were assessed at 130 local clinical study sites in the United States, Canada, and Puerto Rico during the controlled trial phase, with a followed up by telephone from a centralized location during the observational phase (Kryscio et al. 2013). Unfortunately, results from this American study cannot be generalized to European populations with lower Se levels.

## Concluding remarks

Accumulated evidence indicates that AD neuropathology involves multiple biological pathways. The amyloid cascade hypothesis has dominated the field for over 20 years, resulting in a large number of studies with focus on approaches to inhibit formation of and remove A*β* from senile plaques. Yet these trials have failed to demonstrate significant cognitive improvements in patients. Thus novel pharmacotherapies should not be limited to the postulate of the amyloid cascade hypothesis alone, since events occurring intracellularly may prove to be more important for an understanding of the pathology of AD.

Selenate can act as potent inhibitor of tau hyperphosphorylation, presumably by optimizing the functions of antioxidative selenoenzymes that protect the neuronal cytoskeleton. Several nutritional and life-style factors may be involved in AD progression and prevention and the preventive roles of intracellular selenoenzymes against derangement of microtubules and neuronal integrity in the hippocampal area are emphasized in this review. A prophylactic role of optimized Se intake is suggested. Primary prevention should aim at an adequate nutritional intake of Se securing optimal expression of selenoproteins.

A *secondary prevention trial* using Se supplementation at higher doses, e.g. 200 μg/day, to overcome extracellular Se trapping by Aβ in the brain, is of particular importance. Selenized yeast has been used in several clinical trials, although selenate, the primary species used in animal models, is also well tolerated and passes the blood brain barrier (Corcoran et al. 2010a). The intervention should be directed towards elderly with diagnosed MCI. in European regions with low baseline Se intake (Fig. 2), i.e. in Sweden and Norway.
